# Comparison of safety and tolerability with continuous (exenatide once weekly) or intermittent (exenatide twice daily) GLP-1 receptor agonism in patients with type 2 diabetes

**DOI:** 10.1111/j.1463-1326.2012.01639.x

**Published:** 2012-07-19

**Authors:** T Ridge, T Moretto, L MacConell, R Pencek, J Han, C Schulteis, L Porter

**Affiliations:** 1 American Health NetworkIndianapolis, IN, USA; 2 Amylin Pharmaceuticals, Inc.San Diego, CA, USA

**Keywords:** adverse event, exenatide once weekly, exenatide twice daily, safety, tolerability

## Abstract

**Aims:**

Exenatide is a glucagon-like peptide-1 receptor agonist shown to improve glycaemic control in patients with type 2 diabetes (T2DM). Intermittent exenatide exposure is achieved with the twice-daily formulation (ExBID), while the once-weekly formulation (ExQW) provides continuous exenatide exposure. This integrated, retrospective analysis compared safety and tolerability of ExQW vs. ExBID in patients with T2DM.

**Methods:**

Data were pooled from two open-label, randomized, comparator-controlled, trials directly comparing ExQW (N = 277) to ExBID (N = 268). Between-group differences in adverse event (AE) and hypoglycaemia incidences were calculated. Incidence over time and duration of selected AEs (nausea, vomiting, and injection-site-related AEs) were also summarized.

**Results:**

The most common AEs were nausea, diarrhoea, injection-site pruritus, and vomiting. Nausea and vomiting occurred less frequently with ExQW vs. ExBID, peaking at initiation (ExQW) or at initiation and dose escalation (ExBID), and decreasing over time. Few patients discontinued because of gastrointestinal-related AEs. Injection-site AEs were more common with ExQW but decreased over time in both groups. No major hypoglycaemia occurred; minor hypoglycaemia occurred with low incidence in patients not using concomitant sulphonylurea, with no difference between ExQW and ExBID. Serious AEs and discontinuations because of AEs were reported with similar frequency in both groups.

**Conclusions:**

Both exenatide formulations were generally safe and well-tolerated, with ExQW associated with less nausea and vomiting but more injection-site AEs. Continuous vs. intermittent exposure did not impact the overall tolerability profile of exenatide, with no evidence of prolonged duration or worsened intensities of AEs with continuous exposure.

## Introduction

Type 2 diabetes mellitus (T2DM) is a chronic disorder characterized by a dysfunction in glucose regulation leading to hyperglycaemia. The glucagon-like peptide-1 (GLP-1) receptor agonist class of drugs has been showed to improve glycaemic control by coordinating multiple mechanisms of action including induction of glucose-dependent insulin secretion, inhibition of glucagon secretion, enhancement of satiety, and slowing of gastric emptying [1–7]. Thus, GLP-1 receptor agonists act on several systems to modulate plasma glucose concentrations.

Exenatide is a subcutaneously injected, peptide GLP-1 receptor agonist that has been shown to improve glycaemic control, promote weight loss, and improve some cardiovascular risk markers in patients with T2DM [8,9]. The two formulations of exenatide, exenatide once weekly (ExQW) and exenatide twice daily (ExBID), both approved for the treatment of T2DM in the US and Europe, provide continuous or intermittent GLP-1 receptor activation, respectively. ExQW encapsulates the exenatide molecule of ExBID into poly-(d,l-lactide-co-glycolide) microspheres, allowing a gradual rise in exenatide plasma concentration as it is released via diffusion from the biodegradable microspheres [10]. With weekly dosing, this formulation reaches minimally effective therapeutic concentrations of exenatide within 2 weeks and steady state concentrations providing continuous exposure to exenatide by about 6–7 weeks [9,11,12]. In contrast, the ExBID formulation is administered as a bolus injection prior to the two largest meals of the day and has a systemic half-life of 2.4 h [13].

Two open-label, randomized, controlled, clinical studies directly compared the efficacy, safety and tolerability of the two formulations of exenatide in patients with T2DM over 24 or 30 weeks of treatment. ExQW was showed to be superior to ExBID in reducing haemoglobin A1c (HbA1c) over 24 or 30 weeks [11,14]. In these studies, least squares (LS) mean changes from baseline in HbA1c were −1.9% (ExQW) and −1.5% (ExBID) 11 and −1.6% (ExQW) and −0.9% (ExBID) [14], with significant LS mean treatment differences of 0.33 and 0.67%, respectively. While both formulations reduced both fasting and postprandial glucose, ExQW had significantly greater effect on fasting glucose than ExBID whereas ExBID had significantly greater effects on postprandial glucose than ExQW. Patients in both treatment groups lost similar amounts of weight in the two studies. Few differences in the safety and tolerability of the two formulations were observed in the individual studies [11,14].

The goal of this retrospective integrated analysis was to characterize the comparative safety and tolerability of the extended-release (ExQW) and immediate-release (ExBID) formulations of exenatide using the pooled data from the two head-to-head pivotal trials [11,14]. This analysis was performed to increase the likelihood of detecting and characterizing differences in the onset, incidence, or duration of adverse events (AEs) between the two formulations.

## Materials and Methods

### Study Participants and Procedures

Patients from two randomized, controlled, open-label, studies of similar design (DURATION-1 and DURATION-5) were included in this analysis. The population included 545 intent-to-treat (ITT) patients (277 ExQW; 268 ExBID) with T2DM treated with diet/exercise and/or up to 2 oral antidiabetic medications [any combination of metformin, sulphonylurea (SU) or thiazolidinedione (TZD)]. Patients were randomized to receive 2 mg of ExQW or 5 µg of ExBID for 4 weeks followed by 10 µg ExBID for the remainder of the 24- or 30-week study (all patients in DURATION-1 received 5 µg of ExBID for 3 days prior to randomization) 11. Patients were at least 18 years of age and had a baseline HbA1c of 7.1-11.0%, a body mass index of 25–45 kg/m^2^, and stable body weight for at least 3 months prior to the screening visit. Patients were excluded from the studies if they had evidence of a clinically significant medical condition or had regularly used systemic corticosteroids, alpha-glucosidase inhibitors, or meglitinide.

Patients self-administered exenatide during the trial and data was collected at scheduled visits that occurred at weekly, 4-week, or 6-week intervals. The occurrence of AEs was reported spontaneously by the patient or noted by the investigator. Documented AEs were attributed to a defined period according to the event onset date. The intensity of an AE (mild, moderate or severe) was assessed by the investigators according to predefined and standardized definitions. Specific details of the study procedures, as well as efficacy and safety data, have been reported previously for the individual studies [11,14]. Clinical protocols for each study were approved by an Institutional Review Board in accordance with the principles described in the Declaration of Helsinki including all amendments through the Seoul revision of 2008 [15]. Patients provided written informed consent before participation.

### Statistical Methods

Subject disposition and baseline demographic information were summarized by treatment group for the ITT population, which included all randomized patients receiving at least one injection of randomized study medication.

Treatment-emergent adverse events (TEAEs) were defined as AEs occurring for the first time or worsening after the first injection of randomized study medication through the end of the 24- or 30-week controlled study period. Between-group differences in incidence and 95% confidence interval (CI) were calculated for serious adverse events (SAEs), AEs leading to withdrawal, frequently occurring AEs, and AEs of interest including hypoglycaemia. The incidence of AEs of interest, including nausea, vomiting and injection-site AEs (pooled analysis of injection-site pruritus, erythema, urticaria and rash), were summarized over time in 2-week intervals, with a new event defined as onset of the event occurring for the first time in a patient and a recurrent event defined as an event that occurred in the same patient in any of the previous 2-week intervals. An event with a duration that spanned more than one 2-week interval was only recorded in the interval corresponding with event onset. Within each 2-week interval, the percentage of patients with events was calculated using the total number of patients remaining in the trial during the defined period.

Major hypoglycaemia included events that (i) resulted in loss of consciousness, seizure, coma, or other mental status change consistent with neuroglycopenia which resolved after administration of glucagon or glucose or (ii) those that required third-party assistance because of severe impairment in consciousness or behaviour and had a glucose value of ≤54 mg/dl. Minor hypoglycaemia included events that had symptoms consistent with hypoglycaemia and glucose values of ≤54 mg/dl prior to treatment. As hypoglycaemia is more frequently observed when exenatide is used in combination with SU, a subgroup analysis by concomitant SU usage was performed [11,13]. Patients were considered to have concomitant SU use if the patient used SU at any point during the 24- or 30-week controlled study period.

A patient was defined as having treatment-emergent antibodies to exenatide if antibodies were present after the first injection of randomized study medication following absence of antibodies or a missing antibody measurement at baseline, or if the titre increased by at least three dilutions from a detectable measurement at baseline. The incidence of AEs by antibody status was compared in each treatment group.

## Results

### Patient Demographics and Disposition

**Figure 1:**
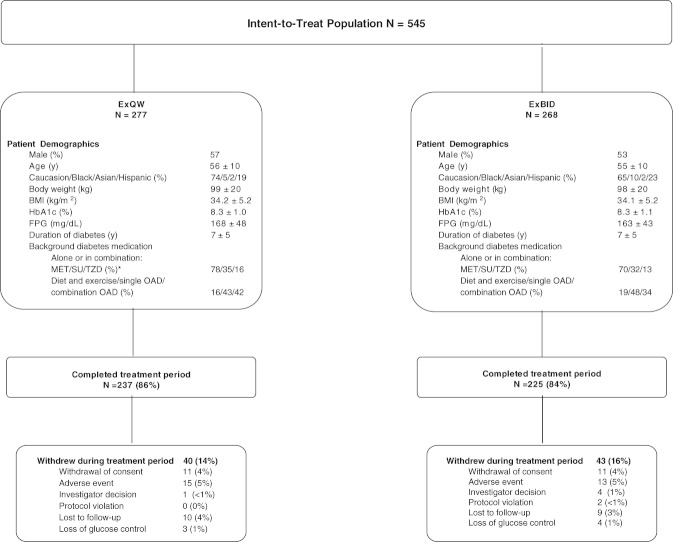
Patient disposition and demographics. Patients were pooled from two open-label, randomized, controlled studies. Patient demographic data are mean ± SD. *Subjects receiving a combination of 2 or more oral antidiabetes medications were included in more than one category. BMI, body mass index; FPG, fasting plasma glucose; MET, metformin; OAD, oral antidiabetes drug; SU, sulphonylurea; TZD, thiazolidinedione.

The ITT population included 545 patients (ExQW N = 277, ExBID N = 268; figure 1). At baseline, patients were either drug-naïve (17%), or treated with one (45%) or a combination (38%) of oral antidiabetes medications (metformin, SU and TZD). Patient demographics were balanced between groups (figure 1), including similar HbA1c (8.3%), fasting plasma glucose (163–168 mg/dl), body mass index (34 kg/m^2^), body weight (98–99 kg), background antidiabetes medications, and duration of diabetes (7 years). Similar numbers of patients withdrew from both groups (14 and 16% with ExQW and ExBID respectively; figure 1). Withdrawals due to AEs occurred in 5% of patients in each group, with <1% of patients withdrawing because of nausea or vomiting (Table 1).

**Table 1:** TEAEs leading to withdrawal

	Treatment		
		
Preferred term	ExQW (N = 277) n (%)	ExBID (N = 268) n (%)	Difference in incidence (ExQW − ExBID) (95% CI)
All AELW	15 (5.4)	13 (4.9)	0.6 (−3.1, 4.3)
Myocardial infarction	1 (0.4)	1 (0.4)	−0.0 (−1.0, 1.0)
Abdominal pain	0	1 (0.4)	−0.4 (−1.1, 0.4)
Diarrhoea	0	1 (0.4)	−0.4 (−1.1, 0.4)
Impaired gastric emptying	1 (0.4)	0	0.4 (−0.3, 1.1)
Nausea	1 (0.4)	4 (1.5)	−1.1 (−2.7, 0.5)
Pancreatitis	1 (0.4)	0	0.4 (−0.3, 1.1)
Regurgitation	0	1 (0.4)	−0.4 (−1.1, 0.4)
Vomiting	1 (0.4)	4 (1.5)	−1.1 (−2.7, 0.5)
Injection-site nodule	1 (0.4)	0	0.4 (−0.3, 1.1)
Injection-site pruritus	1 (0.4)	0	0.4 (−0.3, 1.1)
Malaise	1 (0.4)	0	0.4 (−0.3, 1.1)
Posttraumatic pain	1 (0.4)	0	0.4 (−0.3, 1.1)
Alanine aminotransferase increased	1 (0.4)	0	0.4 (−0.3, 1.1)
Blood creatinine increased	1 (0.4)	0	0.4 (−0.3, 1.1)
Blood potassium increased	1 (0.4)	0	0.4 (−0.3, 1.1)
Lipase increased	1 (0.4)	0	0.4 (−0.3, 1.1)
Weight decreased	1 (0.4)	0	0.4 (−0.3, 1.1)
Anorexia	0	1 (0.4)	−0.4 (−1.1, 0.4)
Paraesthesia	1 (0.4)	0	0.4 (−0.3, 1.1)

AELW, treatment-emergent adverse event leading to withdrawal; CI, confidence interval; ExQW, exenatide once-weekly; ExBID, exenatide twice-daily; TEAEs, treatment-emergent adverse events.

### Treatment-emergent and SAEs

Overall incidence of all TEAEs was similar in patients treated with ExQW (79.4%) and ExBID (76.1%). The majority of AEs were mild or moderate in intensity in both groups.

There was no identifiable pattern of SAEs in either population, with no significant differences observed across System Organ Class AE designations, including cardiac, gastrointestinal (GI), and renal disorders. SAEs occurred with similar incidence with ExQW [4.0% (n = 11)] and ExBID [3.7% (n = 10)], with a non-significant between-group difference of 0.2 (−3.0, 3.5). Two ExQW-treated patients (pancreatitis with no acute inflammatory abnormality which resolved in 3 days while still receiving study medication [14] and fatal myocardial infarction) and one ExBID-treated patient (fatal myocardial infarction) discontinued because of SAEs.

### Adverse Events of Interest

#### GI Adverse Events

**Figure 2:**
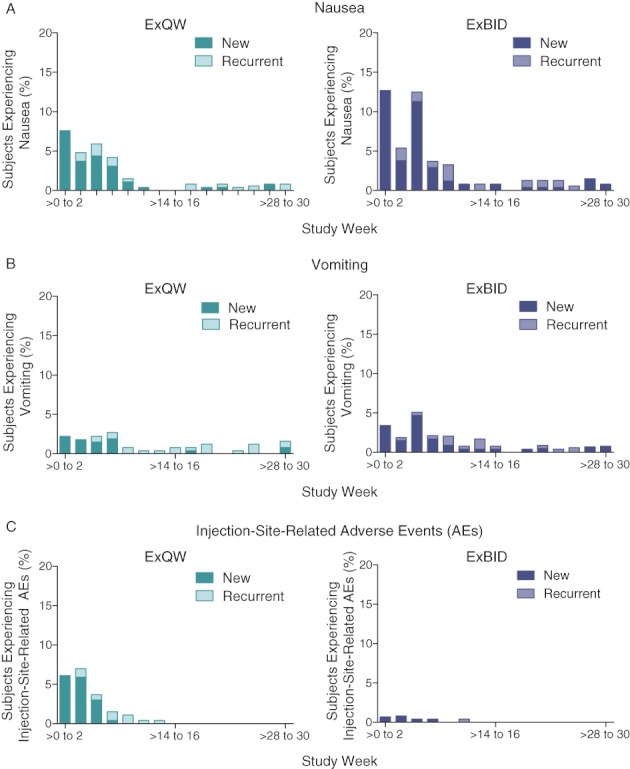
Incidence of new and recurrent nausea, vomiting and injection-site-related adverse events over time and by treatment. Incidence of new and recurrent adverse events (based on time of event onset) through week 30 with ExQW (2 mg; left panel) and ExBID 10 µg (4-weeks at 5 µg, followed by dose increase to 10 µg for the duration of the trial; right panel). Absolute bar height represents entire incidence during a given 2-week interval. (A) Incidence of new and recurrent nausea. (B) Incidence of new and recurrent vomiting. (C) Incidence of new and recurrent injection-site-related adverse events (AEs). Injection-site-related AEs included erythema, pruritus, urticaria, and rash.

The majority of patients in both treatment groups did not experience nausea (79.1% ExQW and 65.3% ExBID) or vomiting (92.1% ExQW and 85.8% ExBID) during the 24- or 30-week study duration. While the overall frequency of GI disorders was not significantly different between ExQW and ExBID groups [−7% (−15, 1.4)], significantly fewer ExQW-treated patients experienced nausea or vomiting compared to ExBID (Table 2). The incidence of nausea and vomiting, as assessed over 2-week intervals, declined over time with continued ExQW or ExBID therapy (figure 2A). The highest incidence of nausea with ExQW (7.6%) occurred at initiation of therapy (within the first 2-weeks). With ExBID, most nausea events occurred during the first 6 weeks of treatment, peaking at initiation of therapy (12.7%) and again between 4 and 6 weeks (12.5%), consistent with the increased ExBID dose at week 4. With continued treatment beyond week 10, nausea occurred in <1% of ExQW- and <2% of ExBID-treated patients to the end of the trial. Nausea recurred in <1% of patients after week 10 for both groups (figure 2A).

**Table 2:** Frequent (≥5%) TEAEs*

	Treatment		
		
Preferred term	ExQW (N = 277) n (%)	ExBID (N = 268) n (%)	Difference in incidence (ExQW − ExBID) (95% CI)
Nausea	58 (20.9)	93 (34.7)	−13.8 (−21.2, −6.3)
Diarrhoea	34 (12.3)	24 (9.0)	3.3 (−1.8, 8.5)
Injection-site pruritus	33 (11.9)	3 (1.1)	10.8 ( 6.8, 14.8)
Vomiting	22 (7.9)	38 (14.2)	−6.2 (−11, −1.0)
Upper respiratory tract infection	21 (7.6)	30 (11.2)	−3.6 (−8.5, 1.3)
Urinary tract infection	19 (6.9)	16 (6.0)	0.9 (−3.2, 5.0)
Injection-site erythema	18 (6.5)	3 (1.1)	5.4 ( 2.2, 8.5)
Constipation	17 (6.1)	14 (5.2)	0.9 (−3.0, 4.8)
Headache	15 (5.4)	17 (6.3)	−0.9 (−4.9, 3.0)
Gastroenteritis viral	15 (5.4)	8 (3.0)	1.1 (−0.5, 2.7)
Dyspepsia	15 (5.4)	6 (2.2)	3.2 (−0.0, 6.4)
Nasopharyngitis	15 (5.4)	9 (3.4)	2.1 (−1.4, 5.5)
Injection-site hematoma	13 (4.7)	22 (8.2)	−3.5 (−7.6, 0.6)
Dizziness	8 (2.9)	17 (6.3)	−3.5 (−7.0, 0.1)

ExQW, exenatide once-weekly; ExBID, exenatide twice-daily; TEAEs, treatment-emergent adverse events.

* All TEAEs with a frequency of ≥5% in either group are listed. (Table includes events assessed as related and unrelated to study drug.)

As with nausea, the highest incidence of vomiting in patients treated with ExBID occurred during treatment initiation and dosage increase and declined over time with continued therapy (figure 2B). Beyond 14 weeks, the occurrence of vomiting in patients treated with ExBID decreased to <1%. ExQW-treated patients exhibited an incidence of vomiting between 1.8 and 2.7% starting at initiation and continuing through week 8 which decreased to 0–1.6% of patients beyond week 8.

Most instances of nausea and vomiting in both groups were mild in intensity and no patients treated with ExQW experienced severe nausea or vomiting. With ExBID, four patients experienced severe nausea and two patients experienced severe vomiting. The duration of nausea was shorter on average with ExQW vs. ExBID. The majority of nausea events, including events of intermittent nausea, were ≤2 days in duration with ExQW and 33% of events resolved in ≤2 days with ExBID. The majority of vomiting events resolved within one day in both ExQW- and ExBID-treated patients.

#### Injection-site-related Adverse Events

The incidence of all injection-site-related adverse events was 22.0% in patients treated with ExQW and 12.7% in patients treated with ExBID with a between-group difference of 9.3% (95% CI: 3.0, 15.6). Two patients discontinued ExQW due to injection-site-related AEs (both mild and resolved) while no injection-site-related withdrawals occurred with ExBID.

The majority of ExQW (84.5%) and ExBID (97.8%) patients did not experience any of the four common injection-site related adverse events (injection-site erythema, pruritus, urticaria, or rash) over the course of the studies. The most commonly reported injection-site-related adverse events were injection-site erythema [6.5% ExQW vs. 1.1% ExBID; between-group difference of 5.4% (2.2, 8.5)] and injection-site pruritus [11.9% vs. 1.1%; between-group difference of 10.8% (6.8, 14.8)]. Other injection-site-related adverse events included injection-site urticaria (0.7% vs. 0.4%) and injection-site rash (1.4% vs. 0.4%), both having no significant between-group differences in incidence rate. The incidence of these injection-site-related events decreased over time in both groups with no events reported beyond 14 weeks in either group (figure 2C). With ExQW, the majority of injection-site-related events resolved within 14 days. With ExBID, the majority (6 of 8) of injection-site-related events required greater than 14 days to resolve. Most events in either group were mild in intensity, with only a single patient treated with ExQW experiencing a severe injection-site adverse event. This patient had an AE of indurated macular rash with severe itching that was deemed non-serious and resolved without patient withdrawal from the study. No patients treated with ExBID reported severe injection-site AEs. Injection-site nodules, primarily transient and mild, were the only other AE reported significantly more frequently with ExQW than ExBID (2.2% vs. 0%) for a between-group difference of 2.2% (0.5, 3.9).

It was noted that although there was no relationship between antibody status and overall incidence of AEs within a treatment group, antibody-positive ExQW-treated patients had a higher incidence of injection-site-related adverse events including injection-site erythema (8.5% vs. 1.4%) and injection-site pruritus (15% vs. 4.2%) compared to antibody-negative ExQW-treated patients. ExBID-treated patients showed no difference in incidence of adverse events by antibody status. Anaphylactic or other systemic immune-related reactions were not observed with either treatment.

#### Hypoglycaemia

No major hypoglycaemia events were reported with either ExBID or ExQW treatments. Patients in either group using concomitant SU had a higher incidence of minor hypoglycaemia events than patients not using a concomitant SU. The incidence of minor hypoglycaemia was similarly low in patients not using concomitant SU in ExQW and ExBID groups (Table 3). There was no apparent pattern in the incidence of hypoglycaemia events over time in either group. There was, however, a trend for less new hypoglycaemia events over time, particularly in patients using concomitant SU.

**Table 3:** Treatment-emergent hypoglycaemia events

	Using concomitant SU agent		Not using concomitant SU agent	
		
Event	ExQW (N = 97) n (%)	ExBID (N = 87) n (%)	ExQW (N = 180) n (%)	ExBID (N = 181) n (%)
Major hypoglycaemia	0	0	0	0
Minor hypoglycaemia	13 (13.4)	14 (16.1)	2 (1.1)	1 (<1)
*Difference (ExQW − ExBID) (95% CI)	−2.7 (−13, 7.6)		0.6 (−1.3, 2.4)	

ExQW, exenatide once-weekly; ExBID, exenatide twice-daily; SU, sulphonylurea.

* Difference calculated for minor hypoglycaemia only.

## Discussion

Efficacy and safety/tolerability profiles for T2DM treatment options are considered in selecting the therapy best-suited to a patient and their particular comorbidities and tolerances [16]. Thus, understanding differences in the safety and tolerability profiles of different agents is a key aspect of making therapeutic decisions.

While ExQW and ExBID contain the same active therapeutic compound, they have different pharmacokinetic profiles, allowing one to remain in the systemic circulation in a continuous manner (ExQW) and the other to provide intermittent exposure over a 24-h period (ExBID). Integrated analyses of the safety and tolerability of intermittent exenatide exposure with ExBID have been previously described [17–19]. In this pooled analysis of two randomized, head-to-head, controlled clinical trials over 24 or 30 weeks of treatment, the safety and tolerability profiles of ExQW and ExBID were directly compared. Both ExQW and ExBID had an equally low incidence of SAEs (4%) and AEs that led to withdrawal (5%). The safety and tolerability profile of ExQW was largely consistent with that of the immediate-release ExBID formulation; there were no indications that continuous exposure to exenatide resulted in an increase in the types, intensity, and duration of AEs observed in the patient [9].

The results of this analysis showed that the primary differences in tolerability between the two therapies were in GI-related and injection-site related AEs. Mild-to-moderate GI-related AEs were the most common AE, with nausea reported most frequently in both groups [11,14]. However, GI tolerability was improved with ExQW vs. ExBID; the incidence of nausea and vomiting was lower with ExQW than ExBID and study withdrawal because nausea occurred less frequently with ExQW than ExBID. Similar to findings in other studies of exenatide or other GLP-1 receptor agonists, the incidence of nausea and vomiting declined over time in both groups, with few reports of GI-related events with longer-term treatment [8,14,20–22]. There was no prolongation in the duration of nausea or vomiting events with ExQW, nor worsened intensity despite the continuous presence of exenatide.

Exenatide-associated nausea and vomiting is thought to be GLP-1-dependent, resulting from slowed gastric emptying, appetite suppression and/or stimulation of neural GLP-1 receptors [23]. While most cases of exenatide-related nausea and vomiting have been reported as mild/moderate in intensity, further mitigation by use of anti-emetics, increased fluid intake, slower eating or smaller meal size has been suggested [23,24]; however, robust data supporting these options is not available. Gradual dose escalation of ExBID has been shown to reduce the proportion of patients experiencing exenatide-related nausea and vomiting [25]. The gradual escalation of exenatide concentrations imposed by the release properties of ExQW may underlie the improved GI tolerability with ExQW compared to ExBID. It is of note that nausea and vomiting are generally not associated with higher concentrations of exenatide. Higher circulating exenatide concentrations, as measured in some individuals administered 2 mg ExQW, did not appear to affect tolerability (Amylin Pharmaceuticals, Inc., data on file). This observation was further supported in the current analysis by the evaluation of GI events over time, in which the highest incidence of nausea and vomiting occurred at initiation, when ExQW levels were at their lowest. A plateau in the incidence of nausea and vomiting was observed as exenatide concentrations continued to increase during the approach to steady state. Thus the decrease in GI events over time, despite the presence of high concentrations of exenatide at steady state, suggests an acclimation to the GI effects of exenatide over time.

Injection-site related AEs occurred more frequently with ExQW compared to ExBID. However, these events were rarely treatment-limiting and few patients (<1%) discontinued as a result of injection-site events. In general, injection-site events were mild and transient, and their frequency diminished over time.

Injection-site nodules were also observed as low-grade foreign body-type reactions occurring in response to the poly-(d,l-lactide-co-glycolide) microspheres that encapsulate exenatide in the ExQW formulation [10,26]. As with other injection-site events, nodules rarely lead to withdrawal and were typically mild and transient in nature.

Consistent with the glucose-dependent mechanism of action of exenatide [27], the overall incidence of hypoglycaemia was low with both ExQW and ExBID treatment and no major hypoglycaemia events occurred. The incidence of minor hypoglycaemia was similar for both groups; however, incidence of minor hypoglycaemia was increased in patients using concomitant SU therapy compared to patients not using SU.

A limitation of this analysis is that it did not include sufficient patient numbers to detect extremely rare AEs and the duration of the trials may not have been long enough to observe AEs that may occur only with extended use of the study drug. Other limitations of this analysis include the open-label nature of the studies and the retrospective analysis of the data.

Overall, continuous (ExQW) vs. intermittent (ExBID) exenatide exposure did not impact the general safety profile of exenatide. This pooled analysis has showed that both exenatide therapies were well-tolerated and resulted in few withdrawals because of AEs. Notably, sustained exenatide concentrations achieved with ExQW resulted in improved GI tolerability and were not associated with a general prolongation or worsened intensity of common AEs.
